# Histopathological Analysis of the Spectrum of Forensic Pathology Lesions Over Two Years From a Tertiary Care Centre in Kerala: A Descriptive Study

**DOI:** 10.7759/cureus.117165

**Published:** 2026-09-29

**Authors:** Bhavya P Mohan, Surekha Vijayan, Jilsa Sucheendran, Sheeju P A

**Affiliations:** 1 Department of Pathology, Government Medical College, Thrissur, Kerala, IND; 2 Department of Forensic Medicine, Government Medical College, Idukki, Kerala, IND

**Keywords:** autopsy spectrum, cardiovascular lesions, forensic pathology, histopathology, public health

## Abstract

Background and purpose: Medicolegal autopsies provide key insights into the causes of death and disease evolution. This study analyzes the spectrum and frequency of histopathological alterations across major organ systems in medicolegal autopsies over two years at Government Medical College, Thrissur, a tertiary care center in South India.

Methods: This descriptive retrospective study included details of 831 autopsy cases. Histopathological findings from cardiovascular, respiratory, hepatobiliary, renal, central nervous, and accessory visceral systems were systematically categorized.

Results: Male predominance was observed (594 cases, 71.5%). The 50-59 (21.1%) and 40-49 (19.3%) age groups were most represented. Evaluated left anterior descending (LAD) arteries (N = 388) frequently showed advanced atherosclerosis (AHA type V: 38.9%, 151 cases). In evaluated hearts (N = 628), hypertrophy was present in 19.1% (120 cases) and healed infarcts in 9.6% (60 cases). Evaluated lungs (N = 676) mostly showed congestion with edema (20.1%, 136 cases), isolated congestion (18.8%, 127 cases), and isolated edema (12.9%, 87 cases). Evaluated livers (N = 476) showed congestion (37.4%, 178 cases) and macrovesicular steatosis (21%, 100 cases). Evaluated kidneys (N = 445) demonstrated congestion (40.2%, 179 cases) and chronic kidney disease (18%, 80 cases).

Conclusion: Histopathological evaluation revealed a significant subclinical burden of chronic cardiovascular, respiratory, and metabolic conditions. Targeted histopathological examination in forensic autopsies enhances diagnostic precision and informs public health statistics.

## Introduction

An autopsy (from Ancient Greek autopsia, "to see for oneself") is a specialized postmortem procedure to establish the cause and manner of death and evaluate a coexisting pathology [1]. Giovanni Morgagni (1682-1771) established clinicopathological correlation (CPC), creating the foundation for modern anatomical pathology [1].

In contemporary medical and legal practice, autopsies are broadly categorized into clinical (pathological) and medicolegal examinations. While clinical/pathological autopsies are requested by treating physicians with the consent of the next of kin to evaluate therapeutic efficacy, assess disease progression, and identify unexpected complications, medicolegal autopsies are mandated by judicial or law enforcement authorities, without requiring family consent, in cases of unnatural deaths, unknown causes of death, or sudden deaths upon hospital arrival, serving to provide essential legal, forensic, and public health data [2,3].

A critical component of comprehensive postmortem evaluation is the integration of histopathology. Gross pathological examination alone frequently falls short of identifying microscopic tissue alterations, cellular damage, or early-stage pathological processes. Beyond the probable cause of death, histopathology uncovers subclinical, incidental, and chronic underlying lesions, such as early myocardial ischemia, microvascular nephropathies, silent hepatic steatosis, or occult malignancies, that remain clinically silent during life [3]. These histopathological insights not only refine the accuracy of determining the cause of death but also contribute significantly to medical literature and education.

Despite the proven value of postmortem tissue analysis, comprehensive data detailing the full spectrum of microscopic visceral findings across large forensic cohorts remains relatively scarce, particularly in developing regions. To address these gaps, this study aimed to test the hypothesis that routine microscopic examination uncovers a high burden of latent, clinically unrecognized chronic pathology in medicolegal autopsies, with the primary objective of systematically defining multi-organ lesion spectra to advance epidemiological surveillance beyond simple descriptive frequency reporting. Unlike previous single-organ or smaller reports, this large two-year cohort study simultaneously maps multi-system histopathological alterations in 831 medicolegal autopsies from South India, providing critical regional data on subclinical disease burdens.

## Materials and methods

Study design and cohort

This retrospective, cross-sectional descriptive study was conducted at Government Medical College, Thrissur, a single tertiary care teaching hospital and referral center in Kerala, South India. The study was carried out over a period of two consecutive years, from January 2020 to December 2021. This retrospective study included all consecutive medicolegal autopsy cases that had available histopathological tissue samples collected over the two-year study period. Cases with severely charred bodies, advanced decomposition, or inadequate tissue preservation resulting in uninterpretable autolytic changes were systematically excluded to ensure diagnostic reliability.

Study population and eligibility criteria

A total of 831 medicolegal autopsies where visceral organs were submitted for histopathological examination were included in this study. Adequately preserved specimens suitable for histopathological examination, with complete demographic and medicolegal records, were included. Cases involving severely decomposed bodies, advanced tissue autolysis, extensive charring, partial autopsies, or specimens with inadequate tissue fixation that precluded reliable microscopic evaluation and incomplete records were excluded from the analysis.

The number of evaluated samples varied across cases due to varying degrees of post-mortem autolysis or focused tissue collection. Consequently, analyses rely on differing denominator subsets. While 831 consecutive cases formed the total study cohort, organ-specific denominator totals varied across different systems. Each table and analysis reflects the exact number of adequately preserved, evaluated specimens available for that specific organ system.

A sample size calculation was not performed using a statistical formula, as a total population (consecutive) sampling strategy was employed. All eligible consecutive autopsy samples during the study period were included to avoid selection bias and provide a comprehensive representation of the data. Data for the study were extracted from the autopsy histopathology registers and requisition forms received in the Department of Pathology, encompassing all eligible consecutive cases during the study period.

Ethical approval and consent

This retrospective study was approved by the Institutional Ethics Committee of Government Medical College, Thrissur, Kerala (IEC/GMCTSR/216/2021, dated 21.10.2021). As the present study involved only the retrospective analysis of archived tissue blocks, slides, and anonymized medicolegal records, the Institutional Ethics Committee waived the requirement for additional individual informed consent. Confidentiality and anonymity of patient and medicolegal data were maintained throughout the study.

Tissue processing and staining

Standardized tissue sampling protocols were consistently applied across all autopsies. Representative tissue blocks were harvested routinely from major organ systems, including the heart (transverse ventricular slices covering left and right ventricles and major coronary artery sections), lungs (multiple lobes), liver (right and left lobes), kidneys (corticomedullary junctions and cortex), brain, and accessory viscera. Tissues were fixed in 10% formalin, processed routinely, including dehydration through graded alcohol concentrations and clearing with xylene. Tissues were subsequently embedded in paraffin blocks. Paraffin-embedded tissue blocks were sectioned at approximately 4-5 µm thickness using a rotary microtome, mounted on glass slides, and stained routinely with hematoxylin and eosin (H&E). Special stains (such as Masson's trichrome or periodic acid-Schiff) were utilized selectively when indicated. 

Histopathological evaluation

Histopathological slides were examined under light microscopy at different magnifications by three experienced pathologists as part of routine clinical diagnosis, and the resulting data were evaluated retrospectively. Any diagnostic discrepancies or borderline cases were discussed among all three pathologists, and a final consensus was reached collectively.

Primary variables included demographics (age, gender) and histopathological lesion distributions across the cardiovascular, respiratory, renal, hepatobiliary, central nervous, and accessory visceral systems.

Histopathological evaluations followed standardized international criteria. Hepatic steatosis was quantified by the percentage of hepatocytes affected by macrovesicular or microvesicular lipid accumulation. Renal lesions and chronic kidney disease markers (such as glomerulosclerosis, tubular atrophy, and interstitial fibrosis) were assessed using standard semi-quantitative grading systems. Coronary atherosclerosis was classified using American Heart Association (AHA) criteria, as outlined below [4]: Type I (initial lesion): characterized by scattered, isolated macrophage foam cells laden with lipid droplets, showing no significant structural remodeling of the arterial wall; Type II (fatty streak): composed predominantly of layers of lipid-laden foam cells (both macrophages and smooth muscle cells) forming visible yellowish streaks, with initial adaptive intimal thickening; Type III (intermediate lesion): represents a transitional phase containing scattered extracellular lipid pools ("extracellular lipid droplets") in addition to foam cell accumulations, subtly disrupting the underlying smooth muscle cell architecture; Type IV (atheroma/core formation): features a distinct, confluent core of extracellular lipid (the lipid-rich necrotic core) that disrupts the normal integrity of the intimal architecture, though without yet forming a thick fibrous cap; Type V (fibroatheroma): advanced lesion characterized by a prominent, well-formed fibrous cap (connective tissue and smooth muscle cells) overlying a deep necrotic lipid core, frequently complicated by calcification, mural thrombosis, and severe luminal narrowing. Type VI (Complicated Lesion): Represents advanced plaques that have undergone structural disruption. It features all the characteristics of Type V lesions alongside surface defects, fissures, hematomas, hemorrhages, or thrombotic occlusion, which frequently trigger acute clinical events like myocardial infarction.

Statistical analysis

Data were collected using Microsoft Excel (Microsoft Corp., USA) and analyzed using IBM SPSS Statistics for Windows, version 25.0 (IBM Corp., Armonk, NY), utilizing descriptive statistics (absolute frequencies and percentages). Continuous variables were summarized using descriptive statistics (absolute frequencies and percentages). Frequencies were reported both relative to evaluated organ specimens and the total cohort (N = 831). To evaluate associations between demographic variables (such as age groups and gender) and specific organ lesions (such as coronary atherosclerosis severity, hepatic steatosis, or chronic kidney disease), inferential statistical analyses including the Chi-square test or Fisher’s exact test were performed where appropriate. A p-value of <0.05 was considered statistically significant.

## Results

Demographic distribution

The cohort comprised 831 cases with a male predominance of 71.5% (n = 594) versus 28.5% female (n = 237) (Table 1). Peak mortality occurred in the 50-59 age group (21.1%, n = 175), followed by 40-49 age group (19.3%, n = 160) and 60-69 (18.8%, n = 156) (Table 2). Pediatric and adolescent cases (<20 years) comprised 8.3% (n = 69). 

**Table 1:** Gender distribution (N = 831)

Gender	Frequency (n)	Percentage (%)
Male	594	71.5%
Female	237	28.5%
Total	831	100.0%

**Table 2:** Age distribution (N = 831)

Age group (years)	Frequency (n)	Percentage (%)
<10	36	4.3%
10–19	33	4.0%
20–29	84	10.1%
30–39	83	10.0%
40–49	160	19.3%
50–59	175	21.1%
60–69	156	18.8%
70–79	77	9.3%
80–89	24	2.9%
>90	3	0.4%
Total	831	100.0%

Cardiovascular spectrum

Coronary artery luminal stenosis (Table 3) and AHA lesion typing (Table 4) were evaluated in submitted arterial segments. Myocardial histology (N = 628 evaluated) showed 226 unremarkable specimens (36% evaluated; 27.2% total) and varied ischemic and structural pathologies (Table 5).

**Table 3:** Coronary luminal stenosis severity RCA: right coronary artery, LAD: left anterior descending artery, LCX: left circumflex artery

Luminal stenosis severity	RCA count	RCA (%)	LAD count	LAD (%)	LCX count	LCX (%)
Patent	140	28.3%	125	25.2%	129	35.7%
5–24%	67	13.5%	56	11.3%	53	14.7%
25–49%	117	23.6%	97	19.5%	68	18.8%
50–74%	94	19.0%	96	19.3%	65	18.0%
75% critical	77	15.6%	123	24.7%	46	12.7%
Total evaluated	495	100.0%	497	100.0%	361	100.0%

**Table 4:** AHA atherosclerosis classification across coronary arteries AHA: American Heart Association

AHA type	RCA count	RCA (%)	LAD count	LAD (%)	LCX count	LCX (%)
Type I	66	17.9%	54	13.9%	43	17.6%
Type II	59	16.0%	61	15.7%	46	18.8%
Type III	65	17.6%	57	14.7%	43	17.6%
Type IV	50	13.6%	61	15.7%	22	9.0%
Type V	123	33.3%	151	38.9%	86	35.1%
Advanced (>V)	6	1.6%	4	1.0%	5	2.0%
Total evaluated	369	100.0%	388	100.0%	245	100.0%

**Table 5:** Spectrum of myocardial lesions (N = 628 evaluated)

Pathological finding	n	% of evaluated (N = 628)	% of the total cohort (N=831)
Unremarkable	226	36.0%	27.2%
Hypertrophy	120	19.1%	14.4%
Vascular congestion	71	11.3%	8.5%
Healed infarcts	60	9.6%	7.2%
Interstitial fibrosis	48	7.6%	5.8%
Myocarditis	37	5.9%	4.5%
Acute infarction	32	5.1%	3.9%
Myofiber disarray	14	2.2%	1.7%
Autolysis	12	1.9%	1.4%
Septic emboli	3	0.5%	0.4%
Infective endocarditis	2	0.3%	0.2%
Neoplasia	2	0.3%	0.2%
Amyloidosis	1	0.2%	0.1%

Respiratory spectrum

Pulmonary histology (N = 676 evaluated) demonstrated high rates of vascular congestion, edema, infectious consolidation, and chronic vascular changes (Table 6).

**Table 6:** Spectrum of pulmonary lesions (N = 676 evaluated) ARDS/DAD: acute respiratory distress syndrome/diffuse alveolar damage

Pathological finding	n	% of evaluated (N = 676)	% of the total cohort (N = 831)
Congestion combined with edema	136	20.1%	16.4%
Isolated vascular congestion	127	18.8%	15.3%
Isolated alveolar edema	87	12.9%	10.5%
Lobar / bronchopneumonia	60	8.9%	7.2%
Intraparenchymal hemorrhage	44	6.5%	5.3%
Active tuberculosis (TB)	36	5.3%	4.3%
Chronic venous congestion (CVC)	36	5.3%	4.3%
Congestion + edema + hemorrhage	35	5.2%	4.2%
Coronary artery disease (CAD)-linked pulmonary effects	20	3.0%	2.4%
Autolysis	19	2.8%	2.3%
Interstitial fibrosis	12	1.8%	1.4%
Emphysema	11	1.6%	1.3%
Pulmonary abscess	11	1.6%	1.3%
Microthrombi in vessels	10	1.5%	1.2%
Primary / metastatic tumors	9	1.3%	1.1%
ARDS/DAD	5	0.7%	0.6%
COVID-19 acute changes	4	0.6%	0.5%
Unremarkable	4	0.6%	0.5%
Unaerated lung	3	0.4%	0.4%
COVID-19 chronic changes	2	0.3%	0.2%
Amniotic fluid embolism	2	0.3%	0.2%
Aerated lung	2	0.3%	0.2%
Actinomycosis	1	0.1%	0.1%

Hepatic spectrum

Liver histology (N = 476 evaluated) revealed vascular congestion, steatosis spectrum, and cirrhotic remodeling (Table 7).

**Table 7:** Spectrum of hepatic lesions (N = 476 evaluated)

Pathological finding	n	% of evaluated (N = 476)	% of the total cohort (N = 831)
Passive sinusoidal congestion	178	37.4%	21.4%
Macrovesicular steatosis	100	21.0%	12.0%
Liver cirrhosis	54	11.3%	6.5%
Macro-microvesicular steatosis	39	8.2%	4.7%
Autolysis	37	7.8%	4.5%
Chronic venous congestion (CVC)	17	3.6%	2.0%
Microvesicular steatosis	13	2.7%	1.6%
Steatohepatitis	13	2.7%	1.6%
Tumors	9	1.9%	1.1%
Necrosis	6	1.3%	0.7%
Cholestasis	4	0.8%	0.5%
Fulminant hepatic failure	3	0.6%	0.4%
Metastasis	2	0.4%	0.2%
Ischemic hepatitis	1	0.2%	0.1%

Renal spectrum

Renal histology (N = 445 evaluated) demonstrated acute circulatory changes alongside chronic renal disease (Table 8).

**Table 8:** Spectrum of renal lesions (N = 445 evaluated) TIN: tubulointerstitial nephritis

Pathological finding	n	% of evaluated (N = 445)	% of the total cohort (N = 831)
Acute congestion	179	40.2%	21.5%
Chronic kidney disease (CKD)	80	18.0%	9.6%
Autolysis	58	13.0%	7.0%
Unremarkable	43	9.7%	5.2%
Acute tubular necrosis (ATN)	40	9.0%	4.8%
Calculous nephropathy	12	2.7%	1.4%
Hyaline arteriosclerosis	10	2.2%	1.2%
Focal segmental glomerulosclerosis	6	1.3%	0.7%
Glomerulonephritis	5	1.1%	0.6%
Acute pyelonephritis	4	0.9%	0.5%
Ischemic necrosis with TIN	3	0.7%	0.4%
Renal tumors	3	0.7%	0.4%
Membranous nephropathy	2	0.4%	0.2%

Central nervous system spectrum

Brain histology (N = 297 evaluated) showed 112 unremarkable cases and prominent acute congestive and ischemic features (Table 9).

**Table 9:** Spectrum of central nervous system lesions (N = 297 evaluated)

Pathological finding	n	% of evaluated (N = 297)	% of the total cohort (N = 831)
Parenchymal congestion	121	40.7%	14.6%
Unremarkable	112	37.7%	13.5%
Cerebral edema	30	10.1%	3.6%
Intracranial hemorrhage	16	5.4%	1.9%
Neoplasia (primary/secondary)	6	2.0%	0.7%
Autolysis	6	2.0%	0.7%
Hypoxic-ischemic encephalopathy	3	1.0%	0.4%
Liquefactive necrosis	2	0.7%	0.2%
Acute meningitis	1	0.3%	0.1%

Accessory visceral systems

Histopathological findings across the remaining evaluated accessory organs are detailed in Table 10.

**Table 10:** Spectrum of lesions across accessory visceral systems

Organ (N evaluated)	Pathological finding	n	% of evaluated	% of the total cohort
Spleen (N = 213)	Unremarkable / unreactive	38	17.8%	4.6%
	Acute congestion	140	65.7%	16.8%
	Chronic venous congestion	19	8.9%	2.3%
	Autolysis	13	6.1%	1.6%
	Congestive splenomegaly	3	1.4%	0.4%
Pancreas (N = 141)	Unremarkable / unreactive	46	32.6%	5.5%
	Autolysis	82	58.2%	9.9%
	Chronic pancreatitis	6	4.3%	0.7%
	Vascular congestion	5	3.5%	0.6%
	Tumors	2	1.4%	0.2%
Skin (N = 36)	Unremarkable / unreactive	12	33.3%	1.4%
	Electrocution burns	12	33.3%	1.4%
	Vascular congestion	8	22.2%	1.0%
	Thermal burns	2	5.6%	0.2%
	Infected ulcerated wound	1	2.8%	0.1%
	Autolysis	1	2.8%	0.1%
Uterus (N = 58)	Unremarkable / unreactive	13	22.4%	1.6%
	Proliferative endometrium	21	36.2%	2.5%
	Secretory endometrium	13	22.4%	1.6%
	Benign leiomyomas	6	10.3%	0.7%
	Tumors	2	3.4%	0.2%
	Postpartum state	2	3.4%	0.2%
	Ruptured vertical scar	1	1.7%	0.1%
Adrenals (N = 79)	Unremarkable / unreactive	22	27.8%	2.6%
	Congestion	47	59.5%	5.7%
	Autolysis	7	8.9%	0.8%
	Hemorrhage	2	2.5%	0.2%
	Adenoma	1	1.3%	0.1%
Stomach (N = 9)	Congestion	3	33.3%	0.4%
	Tumors	3	33.3%	0.4%
	Autolysis	3	33.3%	0.4%
Esophagus (N = 2)	Congestion	2	100.0%	0.2%
Bone marrow (N = 2)	Congestion	2	100.0%	0.2%

## Discussion

This descriptive study underscores the critical diagnostic and public health utility of post-mortem histopathological review. Demographic evaluation revealed a striking male predominance (71.5%, Table 1), with peak frequency occurring between the fourth and sixth decades (Table 2). This concentration among economically active age groups reflects broader South Indian epidemiological trends in sudden and medicolegal deaths [5].

The cardiovascular system demonstrated a substantial silent burden of advanced chronic disease. Critical coronary luminal stenosis (75%), particularly involving the LAD (24.7% of evaluated vessels, Table 3, Figure 1a), highlights a massive reservoir of latent ischemic heart disease, aligning with Association for European Cardiovascular Pathology guidelines [6]. Myocardial remodeling, notably myocyte hypertrophy (19.1% evaluated) and healed infarcts (9.6% evaluated, Table 2), correlates directly with chronic hypertension and long-standing ischemia. 

**Figure 1:**
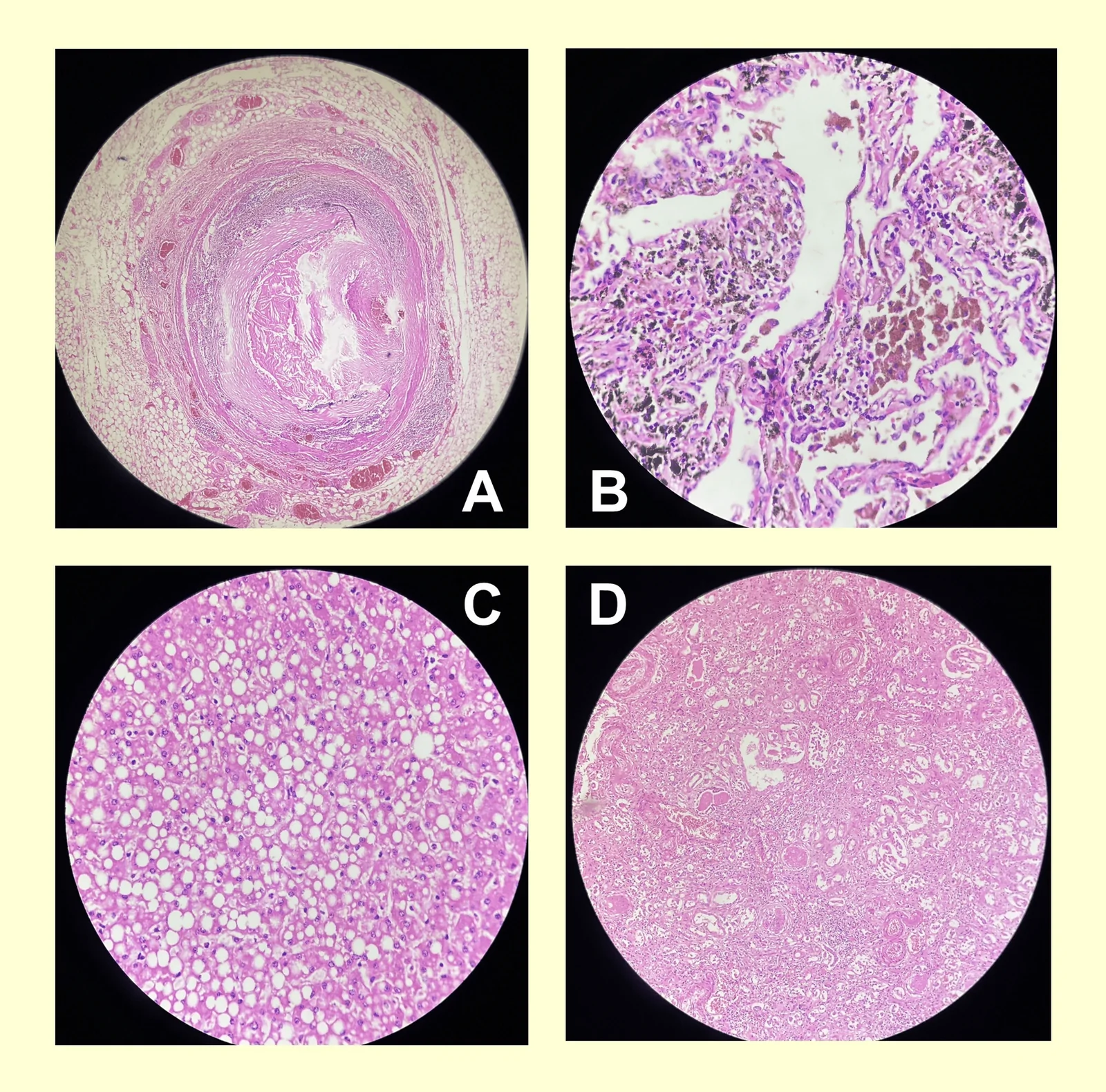
Histopathology images from various organs (H&E stain) (A) Photomicrograph showing coronary atherosclerosis demonstrating plaque causing luminal narrowing with calcification (AHA type V) (H and E x40). (B) Photomicrograph showing chronic venous congestion, lung, characterized by congested capillaries, thickened fibrotic septa, and intra-alveolar heart failure cells (siderophages) laden with hemosiderin pigment resulting from long-standing left-sided circulatory stasis (H and E X400). (C) Photomicrograph showing hepatic steatosis highlighting macrovesicular lipid droplets displacing hepatocyte nuclei (H and E X400). (D) Photomicrograph showing chronic kidney disease depicting global glomerular sclerosis, tubular atrophy, interstitial fibrosis, inflammatory infiltrate and thyroidization of tubules.(H and E x100).

Our findings align closely with regional and international autopsy literature regarding demographic vulnerability and vascular disease burden. Similar to Rao and Sood (2022), our cohort demonstrated a striking male predominance (71.5%) and peak mortality in the fourth to sixth decades, alongside a high prevalence of advanced coronary atherosclerosis [5]. In an urban Indian autopsy series, Patel S et al. (2026) reported cardiovascular involvement in the majority of natural cases with advanced atherosclerosis in 48% [7]. Similarly, Borah KK et al. (2024) and Khare P et al. (2021) established that microscopic evaluation consistently uncovers clinically unrecognized structural lesions, such as macrovesicular steatosis (12-21%) and chronic tubulointerstitial changes that escape macroscopic identification alone [8,9]. Parallel series examining sudden natural deaths similarly identified pulmonary (75%) and coronary (61%) pathology as leading latent causes [10].

Pulmonary pathology was frequent (Table 6), with acute vascular changes (congestion and edema: 20.1% evaluated) representing common final pathways of cardiopulmonary failure. Chronic venous congestion of the lungs was identified in 36 cases, accounting for 5.3% of the evaluated pulmonary specimens and 4.3% of the total cohort of 831 medicolegal autopsies (Figure 1b). Infectious pathologies - bronchopneumonia (8.9% evaluated) and active tuberculosis (5.3% evaluated) - underscore the persisting regional infectious disease burden. In addition, secondary coronary artery disease (CAD)-linked pulmonary changes (3%) represented a crucial etiology-specific subset within the broader spectrum of pulmonary vascular congestion and edema [2,6,10].

Metabolic and vascular modifications heavily predominated across hepatic and renal tissues. Hepatic histology (Table 7) revealed passive sinusoidal congestion as the most common finding (37.4% evaluated), followed by a high prevalence of macrovesicular steatosis (21.0% evaluated) and established liver cirrhosis (11.3% evaluated) (Figure 1c). These findings reflect widespread metabolic syndrome precursors, underlying non-alcoholic fatty liver disease (NAFLD), and chronic alcohol-related injury in the population [11].

Renal evaluation (Table 8) similarly highlighted acute circulatory adjustments dominated by acute congestion (40.2% evaluated), alongside a significant reservoir of structural renal decline marked by chronic kidney disease (18.0% evaluated) and acute tubular necrosis (9.0% evaluated) (Figure 1d). These pathological indicators emphasize deep-seated renal morbidity secondary to systemic insults like chronic hypertension and unmanaged diabetic microvascular damage [12].

Of note, our observation of macrovesicular hepatic steatosis (21% of evaluated livers) and chronic kidney disease (18% of the evaluated kidneys) mirrors the high subclinical prevalence of visceral lesions documented by Borah et al. (2024) [8]. While these microscopic alterations, such as hepatic lipid accumulation and chronic renal structural changes, frequently align with patterns historically linked to metabolic syndrome or chronic hypertension in clinical settings, our forensic dataset lacks direct antemortem clinical histories. Consequently, these findings are more appropriately interpreted as significant morphological manifestations of subclinical organ damage that warrant careful consideration in public health monitoring, rather than definitive proof of specific clinical syndromes.

Central nervous system (CNS) evaluation (Table 9) mirrored terminal systemic events and underlying neuropathologies. Parenchymal congestion and unremarkable histology accounted for 40.7% and 37.7% of evaluated specimens, respectively. Acute structural and hemodynamic alterations included cerebral edema (10.1%) and intracranial hemorrhage (5.4%) secondary to trauma, hypertension, or asphyxia. Neoplasia comprised 2.0%, while hypoxic-ischemic encephalopathy (1.0%), liquefactive necrosis (0.7%), and acute meningitis (0.3%) represented targeted ischemic and infectious insults (Figure 2) [13,14].

**Figure 2:**
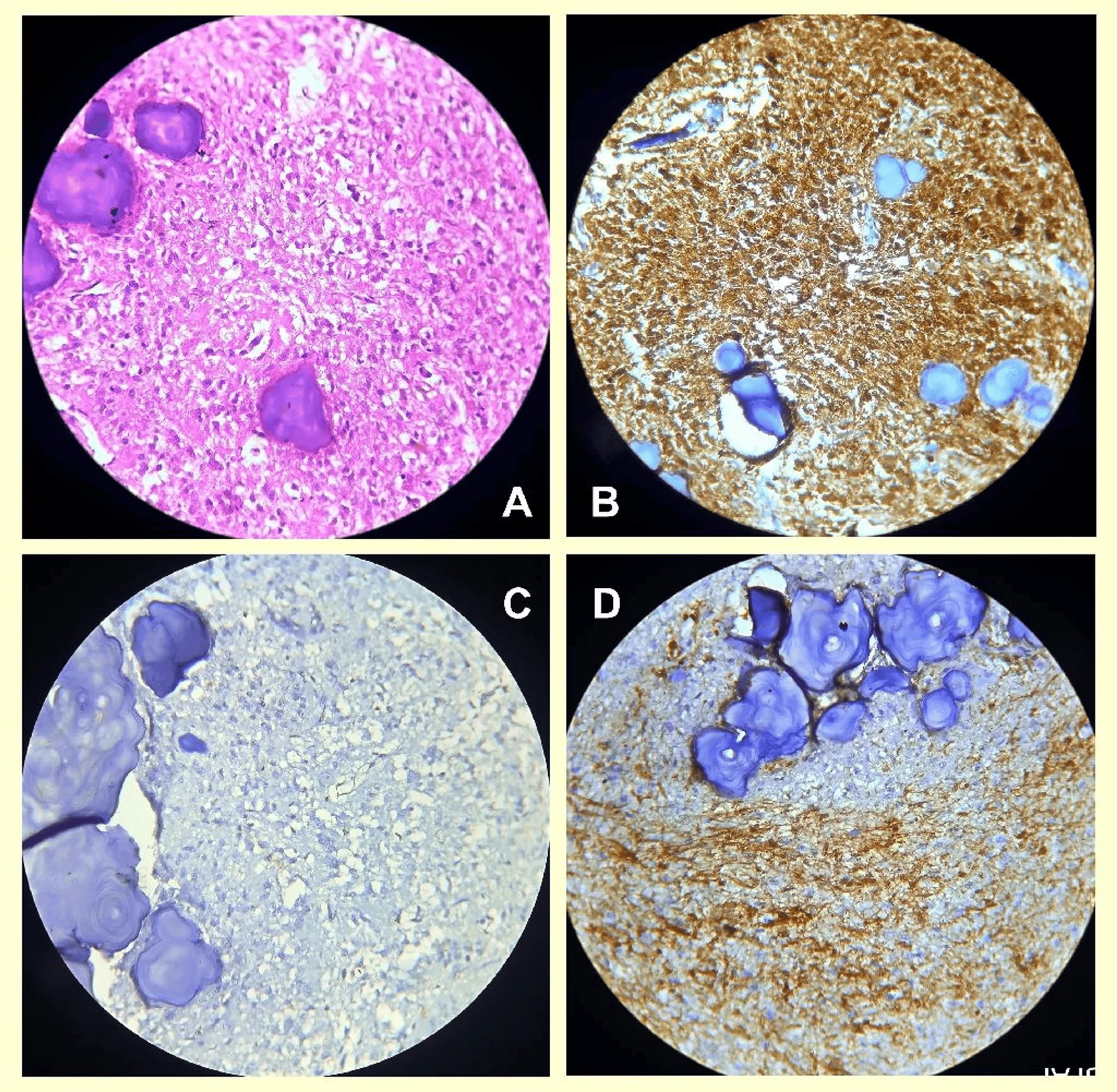
Central neurocytoma (a) Photomicrograph showing a cellular tumor with monotonous round cells with ill‑defined cytoplasm, ovoid nuclei, finely granular chromatin, and micronucleoli, with foci of calcification (H and E ×400). (b) Photomicrograph showing tumor cells showing strong, diffuse NSE positivity (immunostain ×400). (c) Photomicrograph showing tumor cells with EMA negativity (immunostain ×400). (d) Photomicrograph showing GFAP negativity in tumor cells and positivity in reactive astrocytes (immunostain ×400).

Furthermore, evaluation of accessory visceral systems (Table 10) exposed critical subclinical pathology otherwise missed during gross examination. Splenic tissue frequently demonstrated acute congestion (65.7% evaluated) and chronic venous congestion (8.9% evaluated). Pancreatic sections displayed a high frequency of postmortem autolysis (58.2% evaluated), yet concurrently revealed chronic pancreatitis (4.3% evaluated) and localized tumors (1.4% evaluated). Skin specimens highlighted targeted forensic data, capturing electrocution burns (33.3% evaluated) alongside thermal injuries and ulcers. Uterine findings in female cases showed active proliferative (36.2% evaluated) and secretory (22.4% evaluated) phases, alongside benign leiomyomas (10.3% evaluated) and post-partum states. Adrenal glands predominantly exhibited vascular congestion (59.5% evaluated), while limited evaluations of the stomach, esophagus, and bone marrow consistently underscored systemic circulatory collapse through widespread vascular pooling and congestion [13-16].

Routine histopathological examination during medicolegal autopsies provides critical diagnostic validation, uncovers unsuspected co-existing chronic illness, and yields objective data to inform epidemiological monitoring and health system planning [13-16].

Clinicopathological correlation with cause and manner of death

Corroborating microscopic lesions with certified causes and manners of death provides crucial context for interpreting autopsy findings. While macroscopic examination frequently establishes acute triggers of mortality (such as fatal trauma, asphyxia, or acute massive hemorrhage), microscopic evaluation uncovers underlying substrate and contributory pathology. For instance, advanced coronary atherosclerosis (AHA type V) and healed or acute myocardial infarctions frequently substantiate sudden natural deaths categorized under cardiovascular collapse. Similarly, widespread pulmonary congestion and edema serve as final histological common pathways in both cardiac and non-cardiac modes of death, while incidental background findings, such as chronic kidney disease or macrovesicular hepatic steatosis, often represent significant contributory comorbidities that lowered physiological reserve, directly influencing the manner and mechanism of death.

Limitations

This study has several limitations that should be considered when interpreting the results. First, the retrospective single-center design inherently restricts data collection to existing records and archive files, which may limit the generalizability of the findings to other geographical or demographic settings. Second, the absence of detailed antemortem clinical histories prevents definitive correlation between specific microscopic lesions (such as hepatic steatosis or chronic renal changes) and underlying clinical syndromes like metabolic syndrome, hypertension, or diabetes. Third, inconsistent or selective organ sampling across cases means that denominator totals vary across organ systems, potentially introducing selection bias. Fourth, potential interobserver variability in grading histopathological spectrums and atherosclerosis classifications (such as AHA lesion typing) represents an inherent challenge in descriptive pathology. Finally, due to the cross-sectional and postmortem nature of the cohort, the study is unable to establish definitive causal relationships between incidental background pathology and the immediate mechanism or manner of death.

## Conclusions

Systematic histopathological analysis of medicolegal autopsies reveals a high burden of undiagnosed lifestyle diseases within the community. Correlating microscopic findings with certified causes of death highlights how subclinical conditions, such as advanced coronary atherosclerosis, hepatic steatosis, and chronic kidney disease, contribute to mortality. Hence, integrating routine microscopic examination into forensic protocols not only refines the accuracy of cause of death determinations but also transforms forensic pathology into a vital tool for public health surveillance, offering actionable epidemiological data for targeted community health planning for early intervention and disease prevention.

## References

[1] Zampieri F, Zanatta A, Thiene G (2014). An etymological "autopsy" of Morgagni's title: de sedibus et causis morborum per anatomen indagatis (1761). Hum Pathol.

[2] Zarbo RJ, Baker PB, Howanitz PJ (1999). The autopsy as a performance measurement tool--diagnostic discrepancies and unresolved clinical questions: a College of American Pathologists Q-Probes study of 2479 autopsies from 248 institutions. Arch Pathol Lab Med.

[3] Tortosa Lopez JM (2025). Histopathology in medico-legal autopsies: clinical foundation, legal mandate, and judicial guarantee. J Forensic Leg Med.

[4] Stary HC, Chandler AB, Dinsmore RE (1995). A definition of advanced types of atherosclerotic lesions and a histological classification of atherosclerosis. A report from the Committee on Vascular Lesions of the Council on Arteriosclerosis, American Heart Association. Circulation.

[5] Rao D, Sood D, Pathak P, Dongre SD (2014). A cause of sudden cardiac deaths on autopsy findings; a four-year report. Emerg (Tehran).

[6] Basso C, Aguilera B, Banner J (2017). Guidelines for autopsy investigation of sudden cardiac death: 2017 update from the Association for European Cardiovascular Pathology. Virchows Arch.

[7] Patel S, Rajalakshmi BR, Manjunath GV (2016). Histopathologic findings in autopsies with emphasis on interesting and incidental findings-a pathologist's perspective. J Clin Diagn Res.

[8] Borah KK, Thakuria D, Saikia P, Borsaikia K (2024). Spectrum of lung, liver and kidney pathology in autopsy cases: a study at a tertiary center. Int J Res Med Sci.

[9] Khare P, Gupta R, Agarwal S, Bhatnagar A, Anand R (2021). Spectrum of renal lesions on autopsy: experience of a tertiary level institute based on retrospective histopathological analysis. Cureus.

[10] Kulkarni A, Shah VB, Patel R, Janjal S (2025). Evaluation of histopathological findings in sudden unexpected natural deaths (SUND) in adults. Eur J Cardiovasc Med.

[11] Badhan MH, Roy S, Imran J, Alvee MA (2026). The Histopathological Spectrum of Liver Diseases in Medico-Legal Autopsies: Findings From a Tertiary Care Center in Bangladesh. Cureus.

[12] Gujarati SY, Shah PC, Vaghasiya KV, Tandel KC (2023). Histomorphological spectrum of various systems in sudden deaths: an autopsy study at a tertiary care centre in Gujarat, India. J Clin Diagn Res.

[13] Lanjewar DN, Sheth NS, Lanjewar SD, Wagholikar UL (2020). Analysis of causes of death as determined at autopsy in a single institute, The Grant Medical College and Sir J. J. Hospital, Mumbai, India, between 1884 and 1966: a retrospective analysis of 13 024 autopsies in adults. Arch Pathol Lab Med.

[14] Mohan BP, Sivaraman L, Vijayan S, Das AG (2023). Spectrum of incidental tumors in autopsy specimens: a histopathological analysis of 797 cases. Indian J Health Sci Biomed Res.

[15] Zaitoun AM, Fernandez C (1998). The value of histological examination in the audit of hospital autopsies: a quantitative approach. Pathology.

[16] Roulson J, Benbow EW, Hasleton PS (2005). Discrepancies between clinical and autopsy diagnosis and the value of post mortem histology; a meta-analysis and review. Histopathology.

